# Physiologic discrimination of stop consonants relates to phonological skills in pre-readers: a biomarker for subsequent reading ability?^†^

**DOI:** 10.3389/fnhum.2013.00899

**Published:** 2013-12-24

**Authors:** Travis White-Schwoch, Nina Kraus

**Affiliations:** ^1^Auditory Neuroscience Laboratory, Northwestern UniversityEvanston, IL, USA; ^2^Department of Communication Sciences, Northwestern UniversityEvanston, IL, USA; ^3^Institute for Neuroscience, Northwestern UniversityEvanston, IL, USA; ^4^Department of Neurobiology and Physiology, Northwestern UniversityEvanston, IL, USA; ^5^Department of Otolaryngology, Northwestern UniversityChicago, IL, USA

**Keywords:** reading, dyslexia, temporal sampling, phase locking, brainstem, phonological awareness, temporal coding

## Abstract

Reading development builds upon the accurate representation of the phonological structure of spoken language. This representation and its neural foundations have been studied extensively with respect to reading due to pervasive performance deficits on basic phonological tasks observed in children with dyslexia. The subcortical auditory system – a site of intersection for sensory and cognitive input – is exquisitely tuned to code fine timing differences between phonemes, and so likely plays a foundational role in the development of phonological processing and, eventually, reading. This temporal coding of speech varies systematically with reading ability in school age children. Little is known, however, about subcortical speech representation in pre-school age children. We measured auditory brainstem responses to the stop consonants [ba] and [ga] in a cohort of 4-year-old children and assessed their phonological skills. In a typical auditory system, brainstem responses to [ba] and [ga] are out of phase (i.e., differ in time) due to formant frequency differences in the consonant-vowel transitions of the stimuli. We found that children who performed worst on the phonological awareness task insufficiently code this difference, revealing a physiologic link between early phonological skills and the neural representation of speech. We discuss this finding in light of existing theories of the role of the auditory system in developmental dyslexia, and argue for a systems-level perspective for understanding the importance of precise temporal coding for learning to read.

## INTRODUCTION

Learning to read scaffolds on the development of more basic language skills. One such primitive is phonological awareness, the knowledge that spoken language is made up of smaller units such as syllables and phonemes (Sandak et al., 2004; Kovelman et al., 2012; Pugh et al., 2013). Phonological processing has been an area of keen interest in the study of reading for years due to the observation of pervasive performance deficits in dyslexics on basic phonological tasks (Swan and Goswami, 1997; Pugh et al., 2013; Ramus et al., 2013). Theories of developmental dyslexia, and theories of reading more generally, must therefore account for the biological mechanisms supporting phonological processing and related language skills.

Developmental dyslexia affects approximately 5–10% of children and is characterized by a failure to develop effective reading skills despite typical intelligence and adequate support from parents, teachers, and caregivers (Démonet et al., 2004). As a group, children (and adults) with dyslexia have a constellation of deficits in auditory processing. There are, for example, extensive performance gaps between dyslexic and typically developing children on a variety of basic auditory tasks (Wright et al., 1997; Goswami et al., 2002; Ahissar et al., 2006). Children with dyslexia have difficulty coding rapidly changing frequency content in speech such as formant transitions in consonant–vowel syllables (Tallal and Piercy, 1975; Tallal, 1980). Dyslexics also have difficulty tracking amplitude envelope modulations in speech, such as in syllable onsets (Goswami et al., 2002, 2011). However, it remains unknown whether these deficits are each observed within an individual or if there are variable manifestations of developmental dyslexia.

Neurophysiologic deficits associated with dyslexia include increased variability in neural firing as observed in auditory midbrain in humans (Hornickel and Kraus, 2013) and cortex in a rat model of dyslexia (Centanni et al., 2013), in addition to decreased auditory cortical phase-locking to the acoustic envelope (Abrams et al., 2009; Lehongre et al., 2011). Our own group has identified a number of deficits in speech coding throughout the central auditory system that are linked to poor reading (Kraus et al., 1996; Wible et al., 2005; Abrams et al., 2009; Banai et al., 2009; Chandrasekaran et al., 2009; Hornickel and Kraus, 2013).

In light of the wide variety of auditory deficits identified in dyslexics, a plethora of theories as to the disorder’s biological origin have emerged, each of which has tried to identify a “core deficit.” Although these theories are not necessarily mutually exclusive, there is little accord in the literature (cf. Livingstone et al., 1991; Wright et al., 2000; Stein, 2001; Ahissar, 2007; Vidyasagar and Pammer, 2010; Goswami, 2011; Lallier et al., 2013). Many theories have centered on a core deficit in phonological processing and, consequently, a number of neurophysiologic investigations have characterized the biology underlying this skill and deficits thereof. Neuroimaging studies have identified diminished activity in left-lateralized language networks in dyslexic children performing phonological tasks (Kovelman et al., 2012; Pugh et al., 2013). Lehongre et al. (2011) used magnetoencephalography to measure neural entrainment to amplitude modulated noise bursts, and found that dyslexics had poorer phase-locking in the “low gamma” range (~30 Hz), correlating with poor performance on phonological tasks. Finally, the discrimination of stop consonants in auditory midbrain is linked to reading ability in school age children (Hornickel et al., 2009).

While these studies (in addition to many others) have offered insight into the pathophysiology underlying phonological and/or reading deficits, they are complicated by the reciprocal relationship between phonological processing and reading. For although phonological awareness likely bootstraps reading development, the first years of reading themselves influence phonological awareness (Castles and Coltheart, 2004). Therefore, here, we assessed the relationship between phonological awareness and neurophysiologic discrimination of stop consonants in a group of typically developing 4-year-old children. We hypothesized that early phonological awareness is linked to the precision of physiologic speech sound discrimination. To test this hypothesis, we measured neural responses to a pair of speech stimuli previously shown to vary systematically with phonological processing in school-age children (Hornickel et al., 2009). By assessing physiologic processing of speech in pre-school age children we hope to gain insight into the developmental trajectory of reading development. Moreover, we may identify a potential biomarker to predict subsequent reading ability.

## MATERIALS AND METHODS

### SUBJECTS

Four-year-old children (*N* = 26, 14 female) were recruited from the Chicago area to participate in a developmental study at Northwestern University. No child had a history of a neurologic or otologic condition, second language experience, or a diagnosis of autism spectrum disorder. Four children had immediate family histories of dyslexia (parent or sibling). All children passed a brief screening of peripheral auditory function (normal tympanometry and distortion product otoacoustic emissions at least 6 dB above the noise floor for octaves from 1–8 kHz). Additionally, all children had normal click-evoked auditory brainstem responses (Wave V latency < 6.0 ms, measured by a 100 μs click presented at 80 dB SPL to the right ear at 31.25 Hz).

Although we consider these children too young to have attained fully developed reading skills, and so refer to them as “pre-readers,” we note that many of them may have begun some explicit instruction. We did not formally evaluate their reading skills and acknowledge this as a limitation. Nevertheless, we suggest that our cohort represents children who have either not yet begun to learn to read, or are only in the first stages, and so offers novel insight into the relationship between phonological processing and auditory-neurophysiologic responses to speech early in life.

Parents provided informed consent for their children to participate in the study, and the subjects provided verbal assent. The Institutional Review Board of Northwestern University approved all procedures and children were paid $10/hr for their participation.

### BEHAVIORAL MEASURE – PHONOLOGICAL AWARENESS

Phonological awareness was measured with the Clinical Evaluation of Language Fundamentals Preschool, 2nd edn., phonological awareness subtest (CELF 2; Wiig et al., 2004). The test evaluates a child’s knowledge of the sound structure of the English language and measures a child’s ability to manipulate sound through: compound word and syllable blending, sentence and syllable segmentation, and rhyme awareness and production. Raw scores are computed and were used for analysis. The maximum score is 24, and higher scores correspond to better performance. All children met the age-appropriate “criterion” cutoff, indicating that they are within the range of typically developing children. Therefore our data represent a cohort of children with developmentally appropriate performance on the phonological awareness test but with a large range of variability.

### NEUROPHYSIOLOGY: STIMULI

**FIGURE 1:**
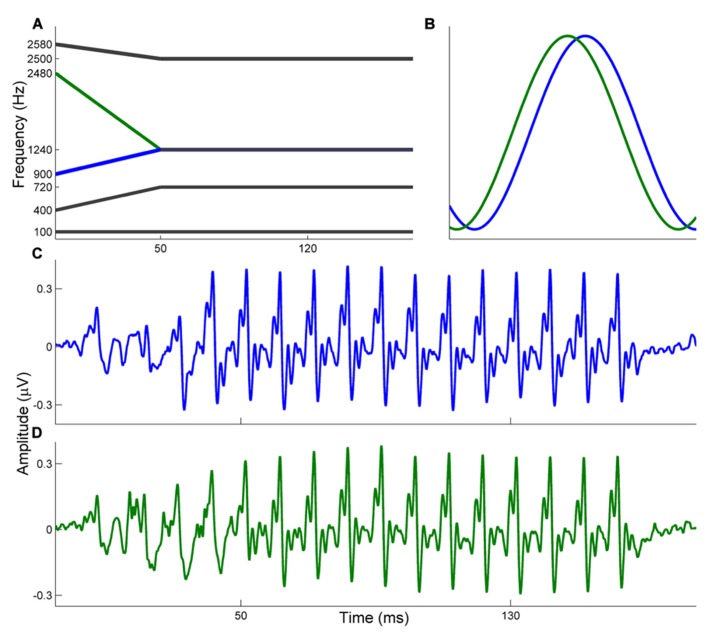
**(A)** Schematic illustrating formant information for the [ba] and [ga] stimuli. The *F*_0_, *F*_1_, and *F*_3_ are identical. The stimuli differ in *F*_2_ onset frequencies with [ba] (blue) ascending and [ga] (green) descending to stabilize for the vowel portion. **(B)** Schematic illustrating the expected phase relationship between brainstem responses to the [ba] and [ga]. Since the [ga] (green) has a higher *F*_2_ onset frequency it is expected that brainstem responses to [ga] phase lead those to [ba]. **(C,D)** Grand average waveforms are displayed for the [ba] (top, blue) and [ga] (bottom, green).

Auditory brainstem responses were elicited in response to the stop consonants [ba] and [ga]. Both consonant–vowel (CV) syllables were 170 ms stimuli that have been described previously (Hornickel et al., 2009). Briefly, both begin with a 5 ms stop burst and have a 50 ms transition from the consonant to the vowel. The vowel is sustained for 120 ms. Both stimuli have a flat fundamental frequency (*F*_0_ = 100 Hz) and during the 50 ms transition the first three formant frequencies shift. The [ba] and [ga] differ only in the *F*_2_ onset frequency (*F*_2OF[ba]_ = 900 Hz; *F*_2OF[ga]_ = 2480 Hz) but are identical in *F*_2_ frequency for the vowel portion (*F*_2VOWEL_ = 1240 Hz; see **Figure 1**). The remaining formants are identical (*F*_1_ = 400–720 Hz; *F*_3_ = 2580–2500 Hz) with *F*_4__-__6_ steady through the 170 ms stimuli (*F*_4_ = 3300 Hz, *F*_5_ = 3750 Hz, *F*_6_ = 4900 Hz). Stimuli were presented monaurally to the right ear at 80.4 dB SPL through electromagnetically shielded insert earphones (ER-3, Etymotic Research, Elk Grove Village, IL, USA). Stimulus presentation was controlled by E-Prime 2.0 (Psychology Software Tools, Inc., Sharpsburg, PA, USA) and stimuli were presented in alternating polarity with an 81 ms interstimulus interval. 4200 sweeps of each stimulus were presented, and the presentation order was randomized for each subject.

### NEUROPHYSIOLOGY: RECORDING AND DATA PROCESSING

Brainstem responses were collected using a BioSEMI Active2 recording system with ABR module. Active electrodes were placed at Cz and each ear with CMS/DRL placed on the forehead, one-half centimeter on either side of Fpz. Only ipsilateral (Cz-A2) responses are used in analysis. Responses were digitized at 16.384 kHz and collected with online filters from 100–3000 Hz (20 dB/decade roll-off) in the BioSEMI ActiABR and recorded into LabView 2.0 (National Instruments, Austin, TX, USA). Since speech-evoked brainstem responses are ideally filtered with a highpass of 70 Hz (Skoe and Kraus, 2010), in MATLAB responses were offline amplified in the frequency domain with an inverse power ramp, 20 dB per decade for 3 decades below 100 Hz (i.e., from 0.1 to 100 Hz, then flat from 0.1 Hz down to DC). Next, a bandpass filter (70–2000 Hz, Butterworth filter, 12 dB/octave roll-off) was applied to frequency-amplified responses. Responses were epoched from -40–213 ms (stimulus onset at 0 ms) and baseline corrected. Artifact rejection was set at ± 35 μVs. Final responses comprised 2000 artifact-free sweeps of each polarity, and responses from alternating polarities were added to emphasize the envelope-following brainstem response while minimizing the influence of stimulus artifact and cochlear microphonic (Campbell et al., 2012).

### NEUROPHYSIOLOGY DATA ANALYSIS: PHASE DISTINCTION BETWEEN RESPONSES

Due to the tonotopicity of the ascending auditory system, stimuli that differ in frequency elicit brainstem responses which are out of phase (Gorga et al., 1988). Therefore, it is expected that responses to [ba] and [ga] begin out of phase from each other (during the transition portion) and are phase-aligned during the vowel, when the stimuli are acoustically identical. In this regard, the relative phases of the two responses are used as proxies for the relative timing of the responses at each frequency. A schematic illustrating this expected relationship is presented in **Figure 1**.

The phase relationship between responses to [ba] and [ga] was measured using custom routines in MATLAB (Skoe et al., in press). Responses were divided into overlapping 20 ms windows from -40–170 ms (1 ms separating each adjacent window) and ramped with a 20 ms Hanning window. The cross-power spectral density function (cspd) was applied between brainstem responses, and power estimates were converted to phase angles to index alignment of the two signals. A larger phase angle (in radians) indicates that the responses are farther out of phase and, therefore, that there is a larger timing lag between responses at a given frequency. A three dimensional “cross-phaseogram” figure is constructed illustrating time (ms, x-axis), frequency (Hz, y-axis), and phase angle (radians, colorbar). During the transition region positive phase angles indicate better neural consonant distinction, as this indicates that [ga] phase-leads [ba], the expected relationship since [ga] has a higher *F*_2OF_.

### SUBJECT GROUPS

Scores on the CELF formed a normal distribution with a mean score of 18.96 (SD, 3.80; Kolmogorov–Smirnov *D*(26) = 0.146, *p* = 0.160). Children were grouped based on their performance on the CELF with a median split defining the top phonological awareness “Top PA” (CELF > 19, *N* = 14, 6 female) and bottom phonological awareness “Bottom PA” (CELF < 19, *N* = 12, 6 female) groups. Five subjects in the Top PA performed at ceiling on the test (scores of 24). Each group included two children with a family history of dyslexia. Groups did not differ in distribution of males and females (*χ*^2^ = 0.154, *p* = 0.70) nor on non-verbal intelligence (matrix reasoning subtest, Wechsler Preschool and Primary Scale of Intelligence, Revised; Wechsler, 1989; *p* = 0.35). As expected, the groups did statistically differ in performance on the CELF, *t*(24) = 9.11, *p* < .001, Cohen’s *d* = 3.56. Summary statistics for the two groups are presented in **Table 1**.

**Table 1:** Demographics for the top and bottom phonological awareness groups are summarized.

	Top PA (*N* = 14)	Bottom PA (*N* = 12)
Males	6	6
Family history of dyslexia	2	2
CELF cutoff	≥20	≤ 18
CELF (raw score)	22.0 (1.7)	15.4 (2.0)
Non-verbal IQ (percentile)	70.8 (24.6)	79.4 (19.9)

*
Groups are matched on all criteria except CELF score. The number of males and number of subjects in each group with a family history of dyslexia are reported. Means (with SDs) are reported for the CELF and for the non-verbal IQ test (Matrix Reasoning sub-test of the WPPSI)
*

## RESULTS

## SUMMARY OF RESULTS

**FIGURE 2:**
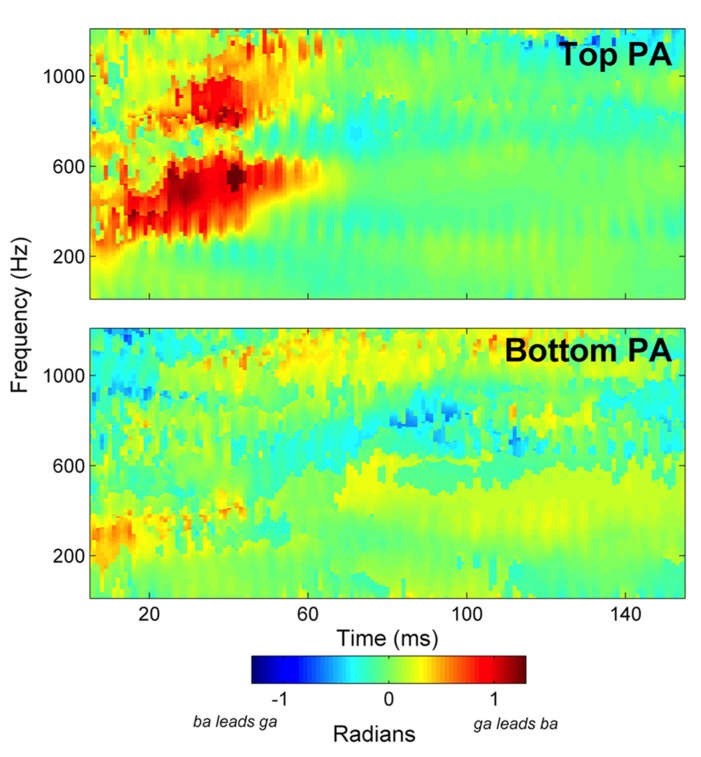
**Group average cross-phaseograms are presented for the Top phonological awareness (PA) and Bottom PA groups.** The Top PA more strongly codes the difference between the [ba] and [ga] stimuli, as indicated by the large red–orange swatches in response to the CV transition.

**FIGURE 3:**
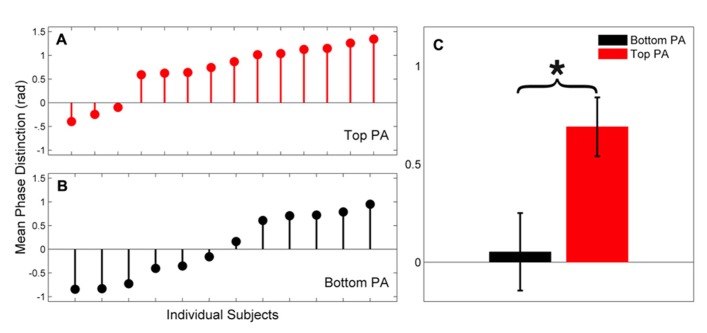
**(A,B)** Phase distinctions for individual subjects (15-55 ms × 300-600 Hz) are presented for the Top PA (Panel A, red) and Bottom PA (Panel B, black) groups. **(C) **Group means are presented. Error bars, ± 1 SEM; *p < 0.05.

Group average cross-phaseograms are presented in **Figure 2**. The Top phonological awareness group (Top PA) evinces a large phase distinction corresponding in time to the transitions in the stimuli, which occurs in the responses from approximately 300–700 Hz (indicated by a large orange–red swatch) and a more moderate phase shift from approximately 750–1000 Hz. Conversely, relatively small phase distinctions were observed in the bottom group (Bottom PA) suggesting that the frequency difference between the stimuli was not strongly represented in these children. Phase distinctions for individual subjects are presented in **Figure 3**, along with group means. No phase distinctions were observed in the response region corresponding to the steady state vowel in either group, as is expected since the stimuli are acoustically identical in the vowel portions.

### PHASE DISTINCTIONS IN THE CONSONANT-VOWEL TRANSITION, 300-700 Hz

Mean phase angle distinctions were calculated for the lower frequency region (15-55 ms × 300-700 Hz). This was the primary region of interest, since it corresponds best to previous reports (Skoe et al., in press; Parbery-Clark et al., 2012). In the Top PA there was a larger mean phase distinction than in the Bottom PA group, *t*(24) = 2.61, *p* = .015, Cohen’s *d* = 1.07. See **Table 2** for descriptive statistics. Since there was a slightly skewed distribution in the Top PA group, this comparison was repeated, and confirmed, with the non-parametric Mann–Whitney *U* test (*p* = 0.015).

**Table 2:** Mean phase distinctions for the two frequency ranges are reported for each group (in rad, with SDs).

		Top PA	Bottom PA
300–700 Hz	Transition	0.690 (0.56)	0.053 (0.69)
	Vowel	-0.041 (0.16)	0.084 (0.56)
750–1000 Hz	Transition	0.551 (0.50)	0.074 (0.74)
	Vowel	-0.075 (0.34)	-0.144 (0.29)

Individual phase distinctions for each group are presented in **Figure 3**. The majority of subjects in the Top PA group had positive phase distinctions whereas most subjects in the Bottom PA group had either very small phase distinctions or distinctions in the opposite of the expected direction (i.e., responses to [ba] phase lead those to [ga]). It is also noteworthy that the magnitude of the largest phase distinctions in the Top PA group exceeds those observed in the Bottom PA group.

Finally, a trending correlation between CELF score and phase distinction was observed (Spearman’s *ρ*(26) = 0.38, *p* = .056) with higher scores on the CELF corresponding to larger phase distinction. We suspect that with a larger subject group, and relatively fewer subjects at ceiling on the CELF, this relationship would be stronger.

### PHASE DISTINCTIONS IN THE CONSONANT-VOWEL TRANSITION, 750-1000 Hz

To further explore group differences, and to ensure that there were no phase distinctions in response to the vowel, additional analyses were pursued. The first analysis focused on the higher frequency phase distinction (25-55 ms × 750-1000 Hz). As indicated in **Figure 2**, the Top PA group had a larger mean phase distinction than the Bottom PA group, *t*(24) = 2.00, *p* = 0.06, Cohen’s *d* = 0.82.

### PHASE DISTINCTIONS IN THE VOWEL REGION

Since the [ba] and [ga] stimuli are identical in the steady state vowel portions no phase distinction is expected in this region. This is reflected in **Figure 2** where green indicates a phase difference of about 0 rad. To confirm this statistically, mean phase angles were calculated for the same frequency regions as in the transition from 60 to 155 ms. There were no group differences in phase distinction for the lower frequency region (300–700 Hz; *t*(24) = 0.81, *p* = 0.427) or higher frequency region (750–1000 Hz, *t*(24) = 0.55, *p* = 0.589).

## DISCUSSION

We assessed the physiologic discrimination of stop consonants in a group of 4-year-old children and reveal a link between this discrimination and phonological awareness. Children with higher phonological awareness had superior neural discrimination of the stop consonants [ba] and [ga], as inferred by far-field electrophysiology. Conversely, children who performed worse on the phonological awareness test, on average, did not robustly distinguish these speech sounds. This relationship has previously been observed in school age children, with neural speech discrimination varying in concert with phonological awareness (Hornickel et al., 2009). By demonstrating this relationship in pre-school children too young to have attained full reading competence we can begin to trace the developmental trajectory of the primitives necessary for complex language-based tasks such as reading. However, we do not know if these children with weak consonant differentiation will soon develop a strong neural differentiation and end up as normal readers, or if they will face challenges as they learn to read. The latter possibility would suggest that these children are at risk for a reading disorder. Regardless, this relationship highlights the role of central auditory processing in developing language skills, and complements phonological deficit theories of reading.

One interpretation of the current results is that they reflect different levels of maturation. The auditory system undergoes rapid developmental plasticity through the first several years of life, and this is reflected in subcortical (Johnson et al., 2008; Skoe et al., in press) and cortical evoked potentials (Choudhury and Benasich, 2011). Individual differences in this rate of maturation may explain the variability in the [ba]-[ga] phase distinction. We do not think this vitiates the link between subcortical auditory function and phonological processing, however. After all, slower maturation of the neural processes important for phonological development may set certain children at a disadvantage when they begin learning to read. Nevertheless, the functional developmental consequences of this maturation for reading (dis)ability remain to be seen.

### TEMPORAL SAMPLING: A SYSTEM-WIDE PERSPECTIVE

Goswami (2011) has proposed a theoretical framework to understand developmental dyslexia, the “temporal sampling framework” (TSF). Under TSF, the core deficit in dyslexia is phonological and is due to impaired oscillatory phase locking for low frequency temporal coding in auditory cortex. An attractive feature of TSF is that it resolves many apparent discrepancies between competing theories of developmental dyslexia. Support for TSF may be found in a large series of psychophysical and neurophysiologic investigations (Witton et al., 1998; Goswami et al., 2002, 2011; Serniclaes et al., 2004; Noordenbos et al., 2012, 2013; Leong and Goswami, 2013; Power et al., 2013).

Although TSF predicts deficient slow cortical phase locking in dyslexia (at rates < 30 Hz; Goswami, 2011), our demonstration of a link between high frequency phase locking in the subcortical auditory system and phonological processing may also be consistent with TSF. While TSF predicts superior *cortical* phase locking at fast rates for dyslexics, and here we report a deficit for high frequency temporal coding in auditory midbrain, we advocate for a systems-level perspective with different “optimal” rates of phase locking as a function of the physiology of the site of interest along the auditory pathway. We see this as compatible with TSF because our view is that the auditory system is best thought of as an integrated circuit that interacts dynamically with cognitive, reward, and other sensory systems (Kraus and Nicol, in press; Kraus and Chandrasekaran, 2010; Bajo and King, 2012; Anderson et al., 2013). The subcortical evoked response we analyzed in the current paper (and others from our group) is generated predominantly by inferior colliculus (IC; for review see Chandrasekaran and Kraus, 2010). Most IC neurons phase lock in the range of 100–1000 Hz (Liu et al., 2006) which is 10-fold the range of the impaired theta and delta oscillatory phase locking in auditory cortex observed in dyslexia (Goswami, 2011). Optimal phonological coding may rely on the interaction of rapid temporal sampling in IC with relatively slow sampling in auditory cortex. Indeed, Abrams et al. (2006) reported that subcortical timing was linked to the temporal integrity of auditory cortical speech coding. Wible et al. (2005) reported correlated subcortical and cortical neural synchrony in representing speech, both of which were diminished in children with language-based learning problems.

That said, relatively little is known about the temporal coding of low frequency information in IC (i.e., < 30 Hz), which may in fact be deficient in dyslexics. Recordings from cat IC do demonstrate phase locking as low as 10 Hz (Langner and Schreiner, 1988), however, the lower limits of phase locking in human IC, and more broadly the oscillatory dynamics of IC, remain an avenue for future research. Temporal coding at multiple rates may occur in parallel through the auditory pathway; evidence from a guinea pig model suggests that a paralemniscal thalamocortical pathway relays slow temporal information to auditory cortex in parallel with fast temporal information relayed through a lemniscal pathway (Abrams et al., 2011). Therefore, a full elucidation of the relationship between auditory phase locking and reading ability on a system-wide level will likely have to accommodate simultaneous temporal coding at multiple rates.

Further support for this integrated view of system-wide temporal coding comes from the rhythm perception literature, which has connected poor reading with an impaired ability to entrain to an external beat and impoverished perception of musical meter (Thomson et al., 2006; Huss et al., 2011; Tierney and Kraus, 2013b). This rhythmic entrainment seems to rely on auditory cortical phase locking (Power et al., 2012). However, the ability to entrain to an external beat is also linked to rapid *subcortical* phase locking and neural synchrony (Tierney and Kraus, 2013a), again suggesting that phase locking across multiple temporal rates may support perceptual skills linked to reading, if not phonological processing itself. An overarching theoretical framework for reading, then, may have to include relatively rapid subcortical phase locking as a key component that interacts with slower cortical oscillatory sampling. Both rapid and slow sampling likely rely on the synchronous firing of neurons in the auditory system, which supports precise representation of transient sounds (McGinley et al., 2012), 0.1 ms precision timing (Anderson et al., 2012), and speech discrimination (Engineer et al., 2008). And once again, dyslexia has been linked to deficits in neural synchrony as observed in humans (Hornickel and Kraus, 2013) and a rat model (Centanni et al., 2013).

This view would be also consistent with the Rapid Auditory Processing theory of developmental dyslexia (RAP; Tallal, 1980; Benasich and Tallal, 2002). Decreased sensitivity to rapidly changing phonological features could drive the impoverished distinction between speech sounds. Previous work has demonstrated that lengthening formant transitions in speech can improve the *cortical* discrimination of speech sounds (Bradlow et al., 1999; Steinschneider and Fishman, 2011), but it is unknown what effect this has on *subcortical* discrimination. Finally, we note that these our findings would be broadly consistent with the view that there are general, non-linguistic sensory deficits in dyslexia (Wright et al., 1997; Stein, 2001; Ahissar et al., 2006). Future work, therefore, should consider the interactions of acoustics, phonemics, and behavioral relevance in subcortical temporal processing.

### A BIOMARKER FOR SUBSEQUENT READING ABILITY?

Although it is important to develop and refine empirically based theories of reading, it is also important to develop methods to identify children at risk for reading disorders. Previous neurophysiologic studies have identified cortical predictors of dyslexia, such as slower right hemisphere polarity shifts in evoked responses to speech (Guttorm et al., 2005; for review, see Leppänen et al., 2012). The structural integrity and volume of left articulate fasciculus is diminished in young children with poor phonological awareness (Saygin et al., 2013). Performance on speech perception tasks is also predictive (Benasich and Tallal, 2002), in addition to oscillatory dynamics in the infant brain (Gou et al., 2011). While the current analysis is not longitudinal, the techniques employed here may one day be useful for predicting future reading ability, either independently or as a complement to existing techniques. In fact, the children in the current study will be tracked over the next several years in hopes of identifying early predictors of subsequent reading ability. We note that the CELF phonological awareness test combines many subskills under the phonological awareness construct (Wiig et al., 2004); it is unknown if group differences are driven primarily by one or two of these subskills, and future investigation is warranted to look specifically at which aspects of phonological awareness are linked to auditory system development.

There are a number of attractive features of the “cross-phaseogram” as a potential biomarker. For one, it is a fast and objective automated procedure. Moreover, as we illustrate here, this measure relates to individual differences in language-based skills. Subcortical evoked responses to speech are relatively easy to obtain and meaningful in individuals (Skoe and Kraus, 2010). From a practical standpoint, responses may be elicited when a child is sleeping or watching a video, thereby eliminating the need for subject compliance in task-related physiologic measurements. And by following the children in this study longitudinally, we may be able to explore individual differences in neurophysiology that distinguish between individual presentations of dyslexia.

### DYSLEXIA, TREATMENT, AND THE SEARCH FOR A CORE DEFICIT

A number of short-term interventions have been employed to improve phonological abilities and reading skills, and these offer further insight into the biological foundations of reading. Some of these studies have focused on perceptual deficits related to poor phonological processing (Tallal et al., 1996; Temple et al., 2003). Other interventions have been broader, such as assistive listening devices that improve classroom signal-to-noise ratios by directing attention to meaningful sound – and in fact also improve neural synchrony in response to speech (Hornickel et al., 2012). Non-speech training such as playing action video games, which improve attentional abilities (Green and Bavelier, 2012), can also improve reading skills (Franceschini et al., 2013), suggesting a role for non-auditory mechanisms in reading development and/or remediation.

Music training, which engenders a host of auditory perceptual and cognitive benefits, may also hold promise. Since precise temporal coding of sound supports fundamental reading skills, and this coding is strengthened by musical experience, it stands to reason that music training may promote the development of reading-related skills (Tierney and Kraus, 2013c). In fact, music experience has been directly linked to improved phonological skills and reading (Moreno et al., 2009; Besson et al., 2011), in addition to physiologic discrimination of speech sounds, as presented in the current study (Parbery-Clark et al., 2012; Strait et al., 2013). Given the established link between rhythm skills and phonological abilities, the rhythmic components of music training may be especially important for developing language-based skills. In fact, Bhide et al. (2013) reported that a comprehensive rhythm training regimen improves phonological skills.

To understand the biological bases of reading, and develop strategies that engender reading skills and remediate dyslexia, it is important to identify which skills to target. In this regard, the quest for the core deficit is important. That said, this search may at times cloud the principal problem, namely, that certain children have tremendous difficulty learning to read. Moreover, the possibility remains that no single deficit accounts for every child who has difficulty reading. Our view is that even without a full understanding of the pathophysiology of dyslexia it is important to identify children at risk as early as possible. Here we have identified a neural correlate of early phonological awareness in pre-school age children. Due to the importance of precise phonological representations for reading this correlate may indicate a biological bottleneck certain children face when they begin to learn to read.

## AUTHOR CONTRIBUTIONS

Travis White-Schwoch and Nina Kraus designed the study; Travis White-Schwoch oversaw data collection and analyzed the data; Travis White-Schwoch and Nina Kraus wrote the paper.

## Conflict of Interest Statement

The authors declare that the research was conducted in the absence of any commercial or financial relationships that could be construed as a potential conflict of interest.
